# German surgeons’ technical preferences for performing total hip arthroplasties: a survey from the National Endoprosthesis Society

**DOI:** 10.1007/s00264-021-05188-x

**Published:** 2021-12-13

**Authors:** Ioannis Stratos, Karl-Dieter Heller, Maximilian Rudert

**Affiliations:** 1 Department of Orthopaedic Surgery, Julius-Maximilians-University Wuerzburg, Koenig-Ludwig-Haus, Brettreichstrasse 11, 97074 Wuerzburg, Germany; 2 Department of Orthopaedic Surgery, Herzogin Elisabeth Hospital, Braunschweig, Germany

**Keywords:** Total hip arthroplasty, Approach, Fast-track-concepts, Minimally invasive surgery, Tranexamic acid, Drain

## Abstract

**Purpose:**

The goal of our study was to conduct an online survey that highlights patterns of practice during total hip arthroplasty (THA).

**Methods:**

The survey was conducted in June and August 2020. Three hundred thirteen members of the German Society for Endoprosthesis participated in the survey.

**Results:**

The anterolateral approach is by far the most popular approach used for primary total hip arthroplasty, followed by the anterior approach during minimally invasive (55% for the anterolateral and 29% for the anterior) and regular surgery (52% for the anterolateral and 20% for the anterior). Two-thirds of the orthopaedic surgeons do not use drainages during THA. Moreover, 80% of the survey participants routinely apply tranexamic acid during surgery. Surgeons who perform minimally invasive surgery for THA use more frequently fast-track-concepts for post-operative rehabilitation. According to the interviewees, the application of fast-track-concepts leads to reduced periods of hospital stay after THA.

**Conclusion:**

Our data demonstrate that patterns of practice during THA in Germany are in line with the evidence provided by current literature. This study can be seen as a stimulus to conduct similar surveys in other countries in order to promote minimally invasive surgery for THA.

## Introduction

Total hip arthroplasty (THA) is a very efficient surgical procedure that provides satisfactory clinical outcomes for decades after primary implantation [1]. Although THA is an established surgical procedure since 1960, there is still a lack of evidence regarding individual steps during surgery and post-surgical care. According to the existing literature, there is no sufficient evidence for superiority of any surgical approach for THA [2, 3]. It has been proven that the use of tranexamic acid (TXA) reduces blood loss and risk of transfusion after primary THA, but no clear evidence exists about routes of administration, dosage, dosing regimen, or time of administration [4]. Although enough literature exists about the benefits of enhanced post-operative recovery with fast-track concepts (FT concepts) after total hip arthroplasty [5], it is unclear how many hospitals use FT concepts in their daily clinical practice.

Surveys are valuable tools in modern evidence-based medicine and can be used to provide insight into current practice and therapeutic approaches for orthopaedic surgery. Data obtained from surveys ultimately design and reshape research. Additionally, polls can further illuminate how deeply research is integrated into the daily surgical practice [6].

The aim of our study was to highlight patterns of practice and surgical preferences during THA in Germany. More specifically, using an online survey, we intended to collect data and to earn insights about surgeons preferred surgical approach (including minimally invasive surgical approaches (MIS)), the usage of a suction drainage (drain), TXA or post-operative FT concepts, and the length of hospital stay during THA.

## Materials and methods

Participants of the survey were members of the German Society for Endoprosthesis (AE; translated from the German “Arbeitsgemeinschaft Endoprothetik”). The AE is part of the German Society for Orthopedics and Trauma Surgery, dedicated to endoprosthesis-related topics. AE members are leading orthopaedic and trauma surgeons and scientists specialized in endoprosthesis and alternative joint-preserving treatment methods [7].

Between June and August 2020, an electronic survey was distributed via email to all 820 AE members. The AE members were anonymously invited to participate in the survey. The survey was administered through an online survey software (Microsoft Forms, Redmond; WA, USA). The invitation email informed about the aim of the survey and provided a link to the survey. By clicking on the provided link, the AE members consented to participate in the survey and agreed to the publication of the results. One and again two weeks after the first invitation, a reminder email was sent.

**Fig. 1:**
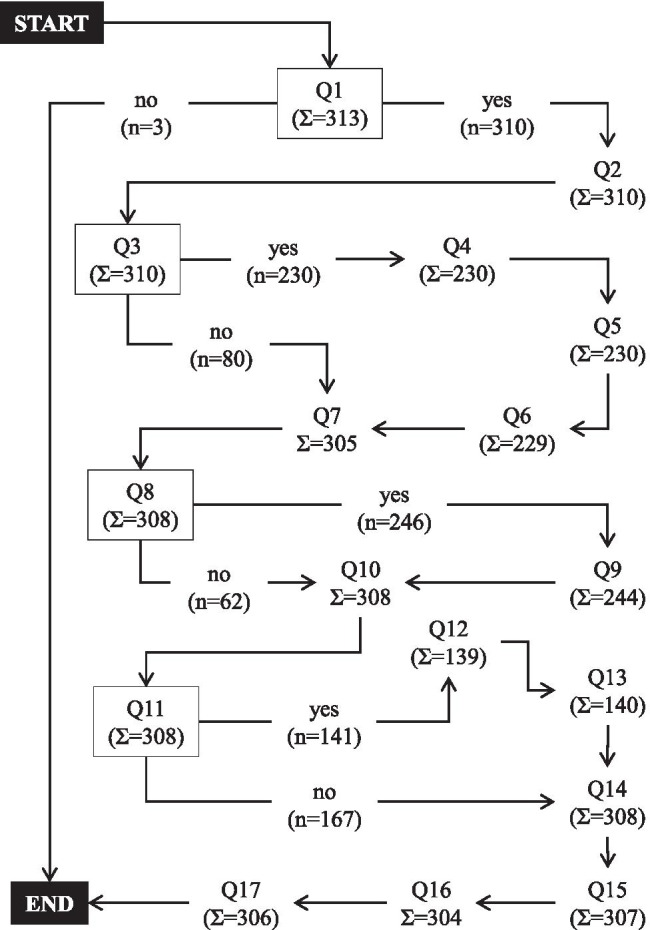
The structure of the survey illustrated as a flowchart. The survey consisted of 17 questions (Q1–Q17). The nodes of the flowchart are marked with rectangles. The sum of responses for each question is given as “Σ,” whereas *n* demonstrates the number of responses for each answer after a node. (Q1, Do you perform THA? Q2, Which surgical approach do you mainly use? Q3, Do you use a minimally invasive approach as your standard surgical approach? Q4, Which minimally invasive approach do you mainly use? Q5, Who uses MIS for THA in your department? Q6, What implants did you use during the introduction of MIS for THA? Q7, Do you routinely use a drainage? Q8, Do you routinely use tranexamic acid? Q9, How do you apply tranexamic acid? Q10, How many THA are performed annually in your department? Q11, Do you use FT concepts for THA? Q12, What kind of FT THA concepts do you use? Q13, Has the introduction of FT THA led to a reduction of the length of hospital stay? Q14, How long is the average length of hospital stay after THA? Q15, Do you perform “outpatient THA”? Q16, Are you planning to perform “outpatient THA” any soon? Q17: how many years have you been working as a surgeon?)

The survey consisted of a maximum of 17 questions (Q1–Q17). All questions are summarized in the legend of Fig. 1 and in Table 1. Depending on the participant’s answers given, the questions varied between 1 and 17. The structure of the survey is illustrated as a flowchart in Fig. 1. The given answers to each question ranged from 1 up to 7. The possible answers to each question are summarized in Table 1.

**Table 1:** Survey’s questions and answers. The survey’s questions (Q1–Q17) are marked in bold. Answers are underlined; “Σ” indicates all given data entries for the question. All answers are given as percentage of Σ (%) followed by the absolute values (in parenthesis). Q5: multiple answers were allowed; *MIS* minimal invasive surgery, *THA* total hip arthroplasty, *FT* fast track

Q1: Do you perform THA? (Σ = 313)	Q9: How do you apply tranexamic acid? (Σ = 244)
	Systemic, 50.4% (123); systemic and local, 40.6% (99); local, 5.3% (13); systemic and local and oral, 2.1% (5); systemic and oral, 0.8% (2); local and oral, 0.4% (1); oral, 0.4% (1)
Yes, 99% (310); no, 1% (3)	Q10: How many THA are performed annually in your department? (Σ = 308)
Q2: Which surgical approach do you mainly use? (Σ = 310)	> 100, 89.0% (274); 50–100, 9.4% (29); 10–50, 1.6% (5)
Anterolateral, 51.9% (161); anterior, 19.4% (60); lateral, 13.9% (43); posterior, 9.0% (28); posterolateral, 3.2% (10); SuperPath, 1.9% (6); other, 0.6% (2)	Q11: Do you use FT-concepts for THA? (Σ = 308)
Q3: Do you use a minimally invasive approach as your standard surgical approach? (Σ = 310)	No, 54.2% (167); yes, 45.8% (141)
Yes, 74.2% (230); no, 25.8% (80)	Q12: What kind of FT THA concepts do you use? (Σ = 139)
Q4: Which minimally invasive approach do you mainly use? (Σ = 230)	Own concepts, 81.0% (113); company supported concepts, 19.0% (26)
Anterolateral, 54.8% (126); anterior, 28.7% (66); posterior, 8.3% (19); lateral, 3.5% (8); posterolateral, 2.6% (6); SuperPath, 2.2% (5)	Q13: Has the introduction of FT THA led to a reduction of the length of hospital stay? (Σ = 140)
Q5: Who uses MIS for THA in your department? (Σ = 230)	Yes: 87.1% (122); no: 12.9% (18)
All senior surgeons, 47.8% (110); some senior surgeons, 41.3% (95); all orthopedic registrars, 31.7% (73)	Q14: How long is the average length of hospital stay after THA? (Σ = 308)
Q6: What implants did you use during the introduction of MIS for THA? (Σ = 229)	4–6 days, 51.6% (159); > 6 days, 44.2% (136); 2–4 days, 2.9% (9); 1–2 days, 1.3% (4)
Standard implants were used, 63.3% (145); new implants were introduced, 16.6% (38); both applied, 20.1% (46)	Q15: Do you perform “outpatient THA”? (Σ = 307)
Q7: Do you routinely use a drainage? (Σ = 305)	No, 99.0% (304); yes, 1.0% (3)
No, 64.9% (198); yes, 35.1% (107)	Q16: Are you planning to perform “outpatient THA” any soon? (Σ = 304)
Q8: Do you routinely use tranexamic acid? (Σ = 308)	No, 93.8% (285); yes, 6.3% (19)
Yes, 79.9% (246); no, 20.1% (62)	Q17: How many years have you been working as a surgeon? (Σ = 306)
	> 20, 60.1% (184); 10–20, 34.3% (105); 0–10, 5.6% (17)

Data analysis and data summarization was conducted by Jamovi v. 1.6.9 (an R-based open-source statistical spreadsheet). To identify differences between group frequencies and group distributions, a Chi-squared test was applied. Statistical significance level was set at 0.05. Statistical analysis was performed using GraphPad Prism v. 9 (GraphPad Software; San Diego, CA, USA).

## Results

In total, 313 out of 820 AE members participated in the survey. This results into a 4.36% margin of error for 95% confidence level assuming a population proportion of 50%. The median number of answered questions for each participant was 15 (minimum, 1 question; maximum, 17 questions). The completion rate for the survey was 98%.

Analysis of the data showed that 99% of the survey participants performed THA (Q1 in Table 1). Ninety-four percent of the interviewees had a surgical experience of at least ten years (Q17 in Table 1), and 89% of the surgeons worked for departments that performed annually over 100 THA (Q10 in Table 1). The average length of hospital stay for a THA was, according to 52% of survey respondents, between 4 and 6 days (Q14 in Table 1). Only three out of 307 surgeons (1%) performed an outpatient THA (Q15 in Table 1), and 6% of all surgeons were interested to offer outpatient THA in the future (Q16 in Table 1).

**Fig. 2:**
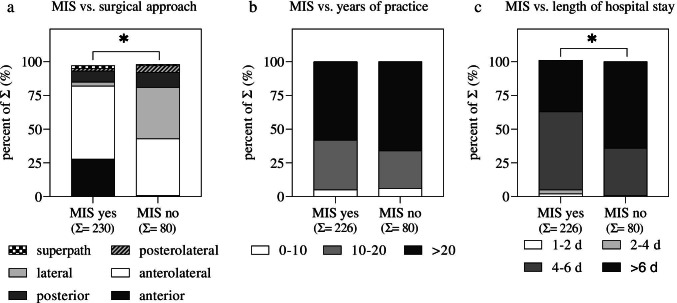
Subgroup analysis between MIS (Q3) and **a** the preferred surgical approach (Q2), **b** years of surgical practice (Q17), and **c** length of declared hospital stay (Q14). Data are shown as percent of the sum (Σ). The Σ of each group is given in the second raw of the *x*-axis; Chi-square test: *p < 0.05. MIS, minimally invasive surgery; for Q2, Q3, Q14, and Q17, see Table 1

With 52%, the anterolateral approach was by far the most popular approach used for primary total hip arthroplasty, followed by the anterior (19%) and the lateral (14%) approach (Q2 in Table 1). Almost three-quarters of all surgeons use a minimally invasive approach as their primary THA approach (230 out of 313; Q3 in Table 1). The most popular minimally invasive approach was the anterolateral approach (55%), followed by the anterior approach (29%) (Q4 in Table 1). The preferred approach significantly differs between surgeons who perform MIS THA compared to surgeons who do not use MIS (Fig. 2a): Statistical analysis using the Chi-squared test showed that the anterior approach is used less frequently and the lateral approach is used more often among surgeons who do not prefer MIS for THA compared to surgeons who do prefer MIS for THA. In approximately half of the departments that can perform minimally invasive surgical approaches for THA, all senior surgeons use MIS THA techniques (Q5 in Table 1). Additionally, 63% of all orthopaedic departments that perform MIS for THA used their standard implants when minimally invasive procedures were firstly introduced (Q6 in Table 1). Further statistical analysis between surgeons who use and surgeons who do not use MIS for THA showed that the surgical experience did not influence surgeons’ decision to perform or not to perform MIS for THA (Fig. 2b). Comparing the declared length for hospital stay between surgeons who perform and do not perform MIS, we could identify a significantly reduced length of hospital stay by the surgeons who perform MIS for THA (Fig. 2c).

**Fig. 3:**
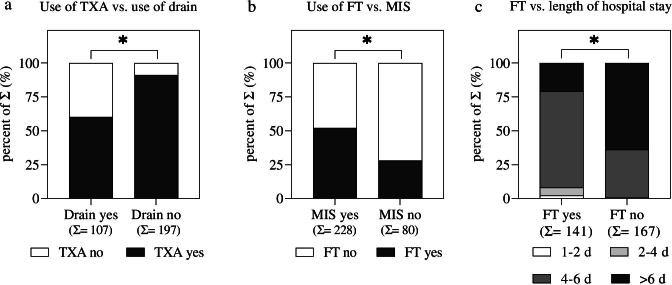
Subgroup analysis between **a** use of TXA (Q8) and use of drain (Q7), **b** use of FT-concepts (Q11) and MIS (Q3), and **c** use of FT concepts (Q11) and length of declared hospital stay (Q14). Data are shown as percent of the sum (Σ). The Σ of each group is given in the second raw of the *x*-axis; Chi-square test: *p < 0.05. MIS, minimally invasive surgery; FT, fast track; TXA, tranexamic acid; for Q3, Q7, Q8, Q11, and Q14, see Table 1

Almost two-thirds of the orthopaedic surgeons do not use drains during THA (Q7 in Table 1). Moreover, 80% of the survey participants routinely apply TXA (Q8 in Table 1) during surgery. Furthermore, most surgeons (91%) administer TXA systemically or systemically and locally (Q9 in Table 1) during THA. We also identified a significant relationship between the use of TXA and drains: Surgeons who apply TXA during surgery are also less likely to use drains compared to surgeons who do not use TXA during surgery (Fig. 3a).

Less than half of the surgeons (46%) use FT concepts for THA (Q11 in Table 1). The company-supported FT concepts are less frequently applied compared to own FT concepts (Q12 in Table 1). Analyzing the relation between FT concepts and MIS for THA, we could identify a significant difference between the groups: Surgeons who use FT concepts have a higher chance of using MIS for THA compared to surgeons who do not use FT concepts for THA (Fig. 3b). According to the declared estimation of the length of hospital stay, 87% of the survey respondents state that FT concepts have led to a reduced length of stay in their hospital (Q13 in Table 1). Analysis between FT concepts and length of hospital stay shows a significant difference between the groups (Fig. 3c): Application of FT concepts leads to shorter periods of declared hospital stay after THA.

## Discussion

The results of this survey provide an insight into current surgical practice during THA in Germany. In summary, the anterolateral approach was by far the most popular approach used for primary total hip arthroplasty, followed by the anterior and the lateral approach. TXA is frequently applied during THA. The minority of surgeons who do not use TXA use drains during THA. Surgeons who perform MIS for THA more frequently use FT concepts for post-operative rehabilitation. Additionally, application of FT concepts leads to shorter periods of hospital stay after THA.

Surveys among physicians appear to have reduced response rates compared to surveys performed in general public [8]. Additionally, survey response rates for physicians are declining over the last years [9]. According to current literature, a usual response rate of a survey addressed to medical doctors ranges between 35 and 45% [8], and studies with response rates below 20% are also found [9]. Taking into consideration the response rate of our study (38.2%), we can conclude that the participation in our survey was above average.

Thirteen years ago, Sendtner et al. performed a similar survey among orthopaedic surgeons in Germany [10] analyzing their surgical practice for total hip arthroplasties. The authors concluded that the posterior and the posterolateral approach were the most popular approaches for non-MIS for THA, whereas the anterior, the anterolateral, and the lateral approach were the most popular approaches for MIS during THA [10]. Considering our current data, we observed a decreased use of the posterior approaches for non-MIS over time, whereas the anterior and the anterolateral approach gained popularity over the last 13 years. Currently, there is no sufficient scientific evidence for a superiority of any THA approach [11]. However, we do believe that one possible explanation for the reduced use of the posterior approach could be its higher dislocation risk when compared to the anterior, lateral, or anterolateral [11].

In the USA, the anterior approach is increasingly becoming an attractive option when it comes to THA. According to a recent publication from Abdel and Berry [12], the most common approach used for THA in the USA is the posterior approach (47%) followed by the anterior approach (40%) and the anterolateral approach (12%). On the contrary, our data suggest that THA is preferably performed in Germany through an anterolateral or lateral approach (66%), whereas the posterior approach is used in only 9% of all cases. As we demonstrated, the anterior approach is used by 19.4% of all surgeons in Germany. The fact that surgeons are less willing to use the anterior approach during THA is multifactorial. Certainly, “incorrect believes” that the anterior approach [13] is associated with limited extensibility during surgery, higher complication rates, increased surgical site infections, and limited use in obese patients are responsible for its reduced use in Germany.

Our data demonstrate that the majority of AE members use TXA during THA. The application of TXA is mainly intravenous or intra-articular, and most surgeons do not use routinely a drain during THA. This pattern of practice is in line with the evidence provided by current literature [14–16]. During post-operative rehabilitation, predominantly non-company-supported FT concepts are used. Additionally, surgeons who use FT concepts declare a reduced period of hospital stay. This fact corresponds to known data from the literature [17].

Outpatient hip arthroplasty is considered to be a safe procedure, that is usually performed on younger, active patients with few comorbidities [18]. Although there is increasing evidence that supports the growing use of “one-day THA” [18], this procedure is not very common in Germany. Our data suggest that only a very small fraction (1%) of all AE members offer outpatient THA. This result is similar to data collected from the USA where approximately 0.7% of all THA are performed on outpatients [19]. One reason that inhibits the performance of outpatient THA is the German hospital billing system. The service “outpatient THA” is not mapped in the DRG [20], and outpatient THA is currently not fully covered by the insurance companies in Germany.

Our data support the assumption that only a few AE members use the posterior, posterolateral, or SuperPath approach during THA. The majority of the AE members use the anterolateral or the anterior surgical approach not only for MIS but also for regular surgery. Unfortunately, the term “minimally invasive surgery” and the term “surgical approach” in THA per se limit the significance of the survey’s answers, mainly because no clear definition for both terms exists. Even authors of meta-analyses for minimally invasive THA [21] have to rely on subjective definitions of MIS from each included scientific article. Some authors define MIS approaches during THA by a short skin incision. Others declare MIS as a muscle sparing or a soft tissue preserving approach [22]. On the contrary, the use of a small surgical incision during THA does not necessarily define an MIS [23]. Additionally, it is very debatable to which extent the lateral approach for a THA can be considered a minimally invasive or muscle sparing approach. The reason for this is that during the lateral approach, the tendon and muscle fibres of the gluteus medius are split at the muscles midway point between the anterior and posterior part [24]. Moreover, the anterolateral approach is not performed uniformly, by all surgeons. In about one out of four papers in recent literature, the term anterolateral approach was used to describe different approaches in terms of anatomy and function [25]. These inconsistencies of the literature make consistent or uniform statements about MIS or surgical approaches difficult to interpret.

By reviewing the literature, two main factors are substantial for the definition of MIS during THA: the length of the surgical incision and the extent of the surgical trauma. In their book, Siebert and Pfeil defined MIS for THA “as a technique which aims to achieve the best preservation of soft tissues and musculature of the hip” [26]. Sendtner et al. define MIS during THA by the incision’s length and the invasiveness of the surgery [10]. In accordance to the current literature and in line with the generally accepted opinion by orthopedic surgeons [10], we believe that MIS for THA should be defined as a surgical technique for THA that is performed (a) through a skin incision just as big as necessary (usually bellow 10 cm) and (b) with the least possible soft tissue injury.

## Limitation

Maybe the biggest limitation of the study lies in the generalizability of the results. Our data are not necessarily transferable to all AE members, all orthopedic surgeons, or even all THA patients in Germany. The lack of random sampling of the AE members, the absence of more survey respondents, and the lack of a trained interviewer who clarifies and reviews the questions can lead to reduced data reliability and reproducibility.

## Conclusions

The anterolateral and the anterior approach are the most commonly used approaches among AE members for THA during standard and MIS. Additionally, the frequency of intra-operative drain or TXA use and the post-operative FT rehabilitation is consistent with the evidence provided by current literature. The current study can be seen as a stimulus to conduct similar surveys in other countries in order to promote MIS and faster recovery after THA.

## Data Availability

The datasets generated during the current study are available from the corresponding author on reasonable request.
